# An explainable artificial intelligence framework for risk prediction of COPD in smokers

**DOI:** 10.1186/s12889-023-17011-w

**Published:** 2023-11-06

**Authors:** Xuchun Wang, Yuchao Qiao, Yu Cui, Hao Ren, Ying Zhao, Liqin Linghu, Jiahui Ren, Zhiyang Zhao, Limin Chen, Lixia Qiu

**Affiliations:** 1 Department of Health Statistics, School of Public Health, Shanxi Medical University, 56 South XinJian Road, Taiyuan, 030001 P.R. China; 2 Shanxi Centre for Disease Control and Prevention, Taiyuan, Shanxi 030012 China; 3 The Fifth Hospital (Shanxi People’s Hospital) of Shanxi Medical University, Taiyuan, Shanxi 030012 P.R. China

**Keywords:** COPD, Machine learning, Class imbalance, Prediction, Smokers

## Abstract

**Background:**

Since the inconspicuous nature of early signs associated with Chronic Obstructive Pulmonary Disease (COPD), individuals often remain unidentified, leading to suboptimal opportunities for timely prevention and treatment. The purpose of this study was to create an explainable artificial intelligence framework combining data preprocessing methods, machine learning methods, and model interpretability methods to identify people at high risk of COPD in the smoking population and to provide a reasonable interpretation of model predictions.

**Methods:**

The data comprised questionnaire information, physical examination data and results of pulmonary function tests before and after bronchodilatation. First, the factorial analysis for mixed data (FAMD), Boruta and NRSBoundary-SMOTE resampling methods were used to solve the missing data, high dimensionality and category imbalance problems. Then, seven classification models (CatBoost, NGBoost, XGBoost, LightGBM, random forest, SVM and logistic regression) were applied to model the risk level, and the best machine learning (ML) model’s decisions were explained using the Shapley additive explanations (SHAP) method and partial dependence plot (PDP).

**Results:**

In the smoking population, age and 14 other variables were significant factors for predicting COPD. The CatBoost, random forest, and logistic regression models performed reasonably well in unbalanced datasets. CatBoost with NRSBoundary-SMOTE had the best classification performance in balanced datasets when composite indicators (the AUC, F1-score, and G-mean) were used as model comparison criteria. Age, COPD Assessment Test (CAT) score, gross annual income, body mass index (BMI), systolic blood pressure (SBP), diastolic blood pressure (DBP), anhelation, respiratory disease, central obesity, use of polluting fuel for household heating, region, use of polluting fuel for household cooking, and wheezing were important factors for predicting COPD in the smoking population.

**Conclusion:**

This study combined feature screening methods, unbalanced data processing methods, and advanced machine learning methods to enable early identification of COPD risk groups in the smoking population. COPD risk factors in the smoking population were identified using SHAP and PDP, with the goal of providing theoretical support for targeted screening strategies and smoking population self-management strategies.

**Supplementary Information:**

The online version contains supplementary material available at 10.1186/s12889-023-17011-w.

## Introduction

Chronic obstructive pulmonary disease (COPD) is a common chronic respiratory disease that is characterized by persistent respiratory symptoms and progressive airflow obstruction. The development of airflow restriction is related to the increased inflammatory reaction of airway passage and lung tissues caused by harmful gases such as tobacco smoke or harmful particles [1]. COPD has become the fourth most lethal disease in the world due to its high morbidity, disability, and mortality [2], imposing a substantial socio-medical-economic burden [3]. The number of individuals with COPD reached 384 million worldwide in 2010, with a prevalence rate of 11.7%, up from 10.7% in 1990 [4]. By 2030, COPD is anticipated to overtake diabetes as the third-leading cause of mortality globally and the seventh-leading cause of morbidity. There are approximately 100 million COPD patients in China, and the prevalence of COPD among people aged 40 years and over is 13.7% [5]. COPD-related deaths accounted for up to 3 million deaths worldwide in 2016, accounting for 5% of all deaths [6]. According to the “China Health and Family Planning Statistical Yearbook”, the number of deaths due to COPD in China reached 876,300 in 2016, ranking third among single diseases and accounting for 9% of total deaths in China. Despite the substantial burden imposed by COPD on health, its screening, diagnosis, and treatment are still insufficient both in China and in other countries. This is because common symptoms, such as fatigue and dyspnoea with exertion, are frequently accepted as normal in elderly individuals [7]. Smokers also commonly accept coughing every morning as a normal occurrence.

Smoking-related mortality, on the other hand, is expected to rise in the coming decades. The WHO estimated that the number of deaths due to tobacco use would increase from 5.4 million in 2005 to 6.4 million in 2015, reaching 8.3 million by 2030 [8]. One reason for this increase is that smoking-induced respiratory changes are typically diagnosed only after respiratory function is impaired. Thus, for the smoking population, new accurate and noninvasive pulmonary function tests are needed.

Moreover, smoking is the primary risk factor for COPD [9], and numerous studies have indicated that smoking promotes the occurrence and progression of a series of pulmonary diseases. For instance, a meta-analysis of 28 studies from 1990 to 2004 and a Japanese study both found that the morbidity of COPD in smokers and ex-smokers was noticeably higher than that in nonsmokers [10, 11]. In 80% of cases, COPD is caused by smoking habits [12], and more than 75% of COPD cases are caused by lung damage due to long-term smoking [13]. Despite the fact that more than 20% of COPD patients have never smoked [14, 15], smoking is not a determinant of the development of COPD and other factors (e.g., second-hand smoke exposure, occupational exposure, and indoor biomass exposure) may also increase the risk of COPD [16, 17]. However, nonsmokers have fewer clinical symptoms, lower levels of inflammatory biomarkers, less airflow limitation and lower airflow exchange impairment, and a lower prevalence of emphysema than smokers [15, 16]. COPD research has continued to focus on the smoking population, and symptomatic smokers and patients with early COPD are most likely to benefit from early treatment. As a result, providing accurate COPD risk predictions for smokers, as well as identifying the factors driving the occurrence of COPD in smokers, can provide a theoretical basis for formulating effective intervention measures in clinical practice.

In recent years, ML algorithms have become an important tool used by clinical workers to facilitate disease detection, diagnosis, and prognosis. More medical practitioners are attempting to apply ML to COPD pathology analysis, clinical diagnosis, and other research [18–25]. However, of the aforementioned COPD-related studies, the majority relied on data sources, including CT scans, genetic biomarkers, lung respiratory sounds, and pulmonary function testing, to conduct risk prediction research for COPD-associated diseases. Due to issues related to data accessibility and cost, most of these studies encountered challenges when attempting to achieve reproducibility at the population level. For instance, reference [18], attempted to differentiate COPD severity levels using respiratory sound data from different channels. The respiratory data were collected by two pulmonologists who used a Littmann 3200 digital stethoscope to simultaneously record data from both the left (L) and right (R) channels in each lung region. However, due to the complexities of data acquisition and evaluation, the sample size was quite limited, with only 6, 5, 5, 5, and 10 cases for the different severity levels, making it challenging to replicate and implement on a larger population scale. Similarly, another study reference [19], introduced a novel approach to nocturnal COPD diagnosis using 44 digital oximetry biomarkers and five demographic characteristics and assessed its performance in a population at risk of sleep-disordered breathing. The study demonstrated good predictive accuracy; however, the ongoing issue remains the limited accessibility of relevant data. Nighttime oxygen monitoring during sleep is typically conducted only for patients with sleep disorders, making it challenging to achieve reproducibility at the population level. Reference [20] employed five clinical features and single-nucleotide polymorphisms (SNPs) to achieve the early prediction of COPD. Reference [22] utilized quantitative CT scans and machine learning to differentiate between COPD and asthma. Both studies faced difficulties in obtaining the amount of relevant data required for model development within large samples from the general population. Furthermore, none of the aforementioned studies on machine learning and COPD have provided comprehensive explanations or analyses of model predictions. Due to the ‘black box’ of machine learning algorithms, it is challenging to determine why specific predictions are made for patients, or, in other words, how specific features of a patient give rise to a certain prediction. To date, the lack of interpretability has constrained the broader application of more powerful machine learning methods to support medical decision making [26], and the limited intuitive understanding of machine learning models remains a substantial hurdle in their implementation in the field of health care [27]. In addition, some studies (excluding case‒control studies and those with a nearly 1:1 ratio of positive to negative samples) have failed to effectively address the issue of data imbalance within their datasets. For instance, in reference [20], the ratio of positive to negative samples in the research data was approximately 1:2. Researchers did not address the existing class imbalance, and further efforts to tackle this imbalance are likely to enhance model performance. Reference [25] evaluated various combinations of CT scan features, texture-based radiomics from CT scans (*n* = 95), established quantitative CT features (*n* = 8), demographic features (*n* = 5), and spirometry measurements (*n* = 3) with machine learning algorithms to predict COPD progression. While the study was comprehensive, it also did not account for dataset imbalance (the dataset imbalance ratio was approximately 1:3).

To address the limitations of previous research, this study comprehensively considered data preprocessing, feature selection, handling of class imbalance in the data, classification models, and model interpretability. We applied a series of data processing techniques and machine learning methods to identify COPD risk groups in the smoking population at an early stage and analysed COPD risk factors in the smoking population using SHAP and PDP methods to support interpretation, aiming to provide theoretical support for targeted screening strategies and self-management of the smoking population. Compared to prior research, the present study has a more comprehensive modelling strategy. Additionally, this study did not incorporate information that is difficult to obtain, such as genetic, imaging, or pulmonary function data. All predictive factors were relatively easy to assess, making them more suitable for widespread application in population screening studies. Furthermore, this study was specifically designed to screen for COPD in the smoking population, a research topic with relatively few studies [28, 29]. It is a targeted screening study for a specific population, with the aim of providing valuable insights into factors influencing COPD and yielding screening models tailored to this group.

## Methods

### Study participants

This survey was based on the 2019 China Resident Chronic Obstructive Pulmonary Disease Surveillance Project and involved a multistage stratified random cluster sampling method. A total of 6648 permanent Chinese residents aged 40 years and older (i.e., who had lived in the survey site for more than 6 months) were surveyed at 11 monitoring sites in Shanxi Province including Taiyuan, Datong, Xinzhou, Linfen, Yangquan, Changzhi, Jincheng, Shuozhou, Jinzhong, Yuncheng, and Luliang. The exact sampling procedure and methods are available in [30]. The Ethical Review Committee of the National Center for Chronic and Noncommunicable Disease Control and Prevention, Chinese Center for Disease Control and Prevention approved this research. All study participants or their guardians signed informed consent forms. All procedures and experiments were carried out according to the applicable rules and regulations.

Residents aged 40 years and older who had lived in the monitored area for more than 6 months out of the previous 12 months and who had daily or occasional active smoking behaviour were eligible for inclusion in this study. Residents who had never smoked were excluded from this study, as were residents living in functional areas (such as sheds, nursing homes, student housing, or military barracks), residents with cognitive or mental disorders, residents with newly discovered and treated cancer, paraplegic individuals, and women who were pregnant or breastfeeding. (A separate word document (see Additional file 1) provides greater detail on the data collection methods and definitions.)

### Dataset

This study distributed surveys to a total of 6648 people. Following data sorting, 841 respondents with missing data (participants without COPD diagnosis results in 2019) were removed, as were 3362 nonsmokers. A total of 2445 participants were included in the study. Of these participants, 378 had COPD, with an imbalance ratio of 5.47, raising the issue of class imbalance. The NRSBoundary-SMOTE resampling technique was used to address this issue. COPD patients were labelled as positive because COPD detection was the focus of this study, whereas non-COPD patients were labelled as negative. A total of 38 variables were selected, including demographic information, respiratory symptoms, smoking status, living environment, cooking and fuel, and occupational exposure. Table 1 and Tables S3 and S4 show the specific variable names and definitions (Additional file 1: Tables S3 and S4).

**Table 1:** Variable assignment, distribution, and missing data

Factors	Assignment (%)	Missing (n)	Missing rate
Current smoking (X_1_)	No = 0(19.5)	0	0.0
	Yes = 1(80.5)		
Use of polluting fuel for household cooking (X_2_)	No = 0(70.5)	569	23.3
	Yes = 1(29.5)		
Use of polluting fuel for household heating (X_3_)	No = 0 (38.0)	64	2.6
	Yes = 1(62.0)		
Occupational exposure to dust and/or hazardous chemical gases (X_4_)	No = 0 (63.2)	0	0.0
	Yes = 1(36.8)		
Pulmonary function (X_5_)	No = 0 (93.9)	0	0.0
	Yes = 1(6.1)		
Awareness of COPD (X_6_)	No = 0(88.0)	0	0.0
	Yes = 1(12.0)		
Education level (X_7_)	Elementary school and below = 1(33.2)	0	0.0
	Junior and senior high school = 2(63.9)		
	College degree and above = 3(2.9)		
Marital status (X_8_)	Single = 1(2.6)	0	0
	Married or cohabiting = 2(91.8)		
	Divorced, widowed, or separated = 3(5.6)		
Family history (X_9_)	No = 0 (79.2)	0	0.0
	Yes = 1(20.8)		
Region (X_10_)	Rural = 1 (71.6)	0	0.0
	Urban = 2 (28.4)		
Sex (X_11_)	Male = 1 (97.6)	0	0.0
	Female = 2(2.4)		
Respiratory disease (X_12_)	No = 0 (87.9)	0	0.0
	Yes = 1(12.1)		
Malignant tumour (X_13_)	No = 0 (99.7)	0	0.0
	Yes = 1(0.3)		
Cardiovascular disease (X_14_)	No = 0 (72.1)	0	0.0
	Yes = 1(27.9)		
Diabetes mellitus (X_15_)	No = 0(94.8)	0	0.0
	Yes = 1(5.2)		
Depression (X_16_)	No = 0 (99.6)	0	0.0
	Yes = 1(0.4)		
Osteoporosis (X_17_)	No = 0 (97.5)	0	0.0
	Yes = 1(2.5)		
Gastroesophageal reflux (X_18_)	No = 0(98.1)	0	0.0
	Yes = 1(1.9)		
Anaemia (X_19_)	No = 0(98.2)	0	0.0
	Yes = 1(1.8)		
Occupation (X_20_)	Agricultural worker = 1 (53.6)	0	0.0
	Nonagricultural worker = 2 (40.9)		
	Retired = 3(5.5)		
Cough (X_21_)	No = 0(90.4)	0	0.0
	Yes = 1(9.6)		
Productive cough (X_22_)	No = 0(82.1)	0	0.0
	Yes = 1(17.9)		
Wheezing (X_23_)	No = 0(93.7)	0	0.0
	Yes = 1(6.3)		
Premature birth (X_24_)	No = 0(97.0)	0	0.0
	Yes = 1(3.0)		
Hospitalization for pneumonia or bronchitis at or before the age of 14 (X_25_)	No = 0(98.7)	0	0.0
	Yes = 1(1.3)		
Hospitalization for pneumonia or bronchitis between the ages of 15 and 17 (X_26_)	No = 0(99.5)	0	0.0
	Yes = 1(0.5)		
Lung surgery (X_27_)	No = 0(99.5)	0	0.0
	Yes = 1(0.5)		
Central obesity (X_28_)	No = 0(30.7)	0	0.0
	Yes = 1(69.3)		
Anhelation (X_29_)	No = 0(87.0)	0	0.0
	Yes = 1(13.0)		
Second-hand smoke (X_30_)	No = 0(22.8)	0	0.0
	Yes = 1(61.7)		
	Unclear = 9(15.4)		
CAT score (X_31_)^a^	Continuous variable	0	0.0
Age, years (X_32_)	Continuous variable	0	0.0
Gross annual income (X_33_)	Continuous variable	2	0.1
Heart rate (X_34_)	Continuous variable	0	0.0
Diastolic blood pressure (X_35_)	Continuous variable	0	0.0
Systolic blood pressure (X_36_)	Continuous variable	0	0.0
BMI (X_37_)	Continuous variable	0	0.0
Size of the premises (X_38_)	Continuous variable	0	0.0
COPD (y)	No = 0(84.2)	0	0.0
	Yes = 1(15.8)		

All participants had to complete the CAT to assess the impact of symptoms associated with pulmonary diseases on their health and daily quality of life

^a^*CAT* COPD Assessment Test

### Data preprocessing

First, samples with excessive missing data or for whom it was impossible to tell whether COPD was present were excluded. Participants and features with low deletion loss rates (< 30%) were retained, and missing values were imputed using factorial analysis for mixed data (FAMD) [31]. According to the results, the loss rates for all features and samples were below 30%. Therefore, only imputation of missing values was performed, and no deletion was applied. Refer to Table 1 for details. Then, min–max normalization was applied, and the one-hot method was used to process multiple classes of variables due to the variety of features and the need to standardize quantitative data for some algorithms, such as SVM [32].

### Feature selection

The redundant information in chronic disease survey data might lead to unsatisfactory classification of COPD in the smoking population, as the excessive dimensionality of the data would reduce the model’s accuracy and efficacy [40]. Therefore, it is crucial to perform feature selection on the raw data using an efficient feature selection method. For example, with random forest as a wrapper algorithm [33], its flexibility in variable selection through various strategies as ‘variable importance measurement (VIM)’ addresses not only the challenge of minimal optimal variable selection but also has an advantage for handling the selection of all relevant variables. It effectively addresses two key issues in selecting all relevant variables: 1) the identification of weakly related variables and 2) the effective differentiation between weak correlations and those caused by random effects [34]. Hence, we opted for a feature selection method based on the random forest algorithm. Furthermore, in 2019, Szymczak’s group analysed the efficacy of multiple RF-based variable selection strategies, such as RFE-RF, Boruta, Altmann, R2VIM, and VIT. After applying a variety of criteria, including sensitivity, the false discovery rate, efficiency, stability, and root mean square error, they found that Boruta and VIT were superior and recommended them [35]. Moreover, Boruta has exhibited encouraging outcomes in various clinical studies, as shown by citations [36, 37]. The researchers conducted model validation and found that the integration of the Boruta algorithm with the classification model demonstrated greater performance than that of LassoCV, SVM-RFE, and Lasso. These findings further emphasize the effectiveness of the Boruta method in the context of feature selection. As a result, we chose the Boruta method [38] based on RF for the feature selection process.

### Class imbalance

In our dataset, the proportion of non-COPD participants was nearly four times that of COPD patients, resulting in substantial class imbalance. Currently, solutions to class imbalance in datasets mainly involve two levels: the algorithm and data levels [39]. The former adds cost-sensitive analysis to some algorithms, with the classes involved in the classification task allocated different misclassification costs [40]. However, determining the best misclassification cost for each class is an enormous project [41]. Methods based on the data level primarily involve resampling techniques. Due to its simplicity and easy implementation, this methodology has been increasingly adopted to address imbalanced datasets [42–44]. In this study, the NRSBoundary-SMOTE resampling method was used to handle imbalanced datasets. This method oversamples the minority class samples in the boundary region. It can broaden the decision space of the minority class samples with little impact on that of the majority class samples [45].

### Prediction models

A support vector machine (SVM) model [46], a logistic regression (LR) model [47], a random forest (RF) model [33], an extreme gradient boost (XGBoost) model [48], a light gradient boosting machine (LightGBM) model [49], a natural gradient boosting (NGBoost) model (NGBoost) [50] and a category boosting (CatBoost) model [51] were developed to predict COPD. To train, construct, and evaluate the seven predictive models, the stratified hold-out test (8:2) was used.

The models were chosen based on several commonly used and advanced types of predictive models. The LR model, RF model, and SVM model, for example, have been widely used in many clinical applications, such as disease prediction in hepatic encephalopathy [52]. In clinical research, the XGBoost and LightGBM models have also been implemented and have demonstrated excellent classification performance [53, 54]. NGBoost is a novel supervised machine learning algorithm that provides probabilistic prediction via gradient boosting with covariate conditioning [50]. CatBoost achieves high accuracy by modifying the gradient to avoid shifting the prediction order. It is capable of handling enormous amounts of information while consuming less memory. It reduces the likelihood of overfitting, resulting in a more generalized model [55]. As a result, these models were chosen to construct predictive models. With the training data, a grid search method with fivefold CV was used to determine the best hyperparameters of the LR, SVM, RF, XGBoost, LightGBM, NGBoost, and CatBoost models. However, tuning parameters were employed in only the LR, SVM, RF, XGBoost, LightGBM, and CatBoost models; the overall performance of the NGBoost model with these parameters was inferior to that with the default settings, so tuning parameters were not used in this model. Table S2 shows all the pertinent parameters.

### Model interpretation

ML models are usually thought of as “black boxes” because it is difficult to explain why an algorithm can yield correct predictions for a specific participant; therefore, we used PDP and SHAP values in the present study. SHAP is an ML model interpretation method proposed by Scott et al. [56] that has both local interpretability and global interpretability. It involves constructing a linear model based on the best “Shapley value” in game theory that can be used to interpret the output of any ML model. It was previously validated for its interpretability [57, 58]. PDP can reflect the marginal effect of features on model prediction [59], as opposed to feature importance, which is the numerical magnitude of the impact of features on the model. Specifically, PDP presents a linear relationship between the impact of features on prediction results and is a model-independent interpretation method. We employed SHAP and PDP to explain our predictive model, which incorporated associated risk factors for COPD in the smoking population. We assessed the significance of the feature ranks from the ultimate model to identify the key predictors of the occurrence of COPD within the smoking population.

### Evaluation parameters

In this study, we used several standard performance indicators, namely, accuracy, specificity, sensitivity, F1-score, G-mean and the area under the receiver operating characteristic curve (AUC), to evaluate the classification performance of the classifiers. These matrices were computed by a binary confusion matrix (Table 2).

**Table 2:** Confusion matrix

Confusion matrix	Predicted	
	Positive	Negatives
Actual		
Positive	True Positive (TP)	False Negative (FN)
Negative	False Positive (FP)	True Negative (TN)

#### Confusion matrix

Each column of the matrix represents the predicted classifications of samples, while each row represents the true classifications of samples. Ultimately, each cell represents one of the possible combinations of predicted and true classifications.

#### Accuracy

This represents the proportion of correctly predicted samples among all the samples with predictions. The calculation formula is as follows:

$$Accuracy=\frac{\left(TN+TP\right)}{\left(TP+TN+FP+FN\right)}\times 100\%$$

#### Specificity

This is the proportion of true negative samples among all the samples predicted to be in the negative class. It measures the model’s ability to identify individuals in the smoking population without COPD. The formula is as follows:

$$Specificity=\frac{TN}{\left(TN+FP\right)}\times 100\%$$

#### Sensitivity

This is the proportion of true positive samples among all the samples predicted to be in the positive class. It measures the model’s ability to identify COPD patients. The formula is as follows:

$$Sensitivity=\frac{TP}{\left(TP+FN\right)}\times 100\%$$

#### F1-score

This is the harmonic mean (a type of average for probabilistic data) and indicates the accuracy of predictions of samples in the positive class. It represents the proportion of samples correctly predicted as positive among those in the positive class. The formula for calculating the F1 score is as follows:

$$F1-Score=\frac{2Precision}{Precision+Recall}\times 100\%$$

#### Area under the ROC curve (AUC)

A comprehensive metric that reflects the magnitude of sensitivity and specificity, typically ranging from 0.5 to 1. A value closer to 1 indicates better predictive performance. The formula for its calculation is as follows:

$$AUC=1-\frac{\frac{FP}{FN+TN}-\frac{FN}{TP+FP}}{2}\times 100\%$$

#### G-mean

The geometric mean of sensitivity and specificity, serving as a comprehensively indicator of the classifier’s ability to correctly identify positive and negative samples. The formula for its calculation is as follows:

$$G-mean=\sqrt{\frac{TP}{TP+FN}\times \frac{TN}{TN+FP}}\times 100\%$$

### Statistical analysis

For statistical analysis, IBM SPSS Version 26 (IBM Corp., Armonk, NY, USA) was used. For data that were normally distributed, the t test was employed; for data that were not normally distributed, the Mann‒Whitney test was used. To compare categorical and parametric variables, the chi squared test or t test/Mann‒Whitney U test was used. The significance levels for all statistical tests were set at *P* < 0.1 (all *P* values were two-tailed). The NRSBoundary-SMOTE resampling method, as well as the development and optimization of all classifier models, was carried out using Python (version 3.9.7). Boruta, a feature dimension reduction method, was constructed in R Studio 4.1.2. (R Development Core Team). The graphs in this article were created in Python (version 3.9.7).

## Results

### Experimental setup

**Fig. 1:**
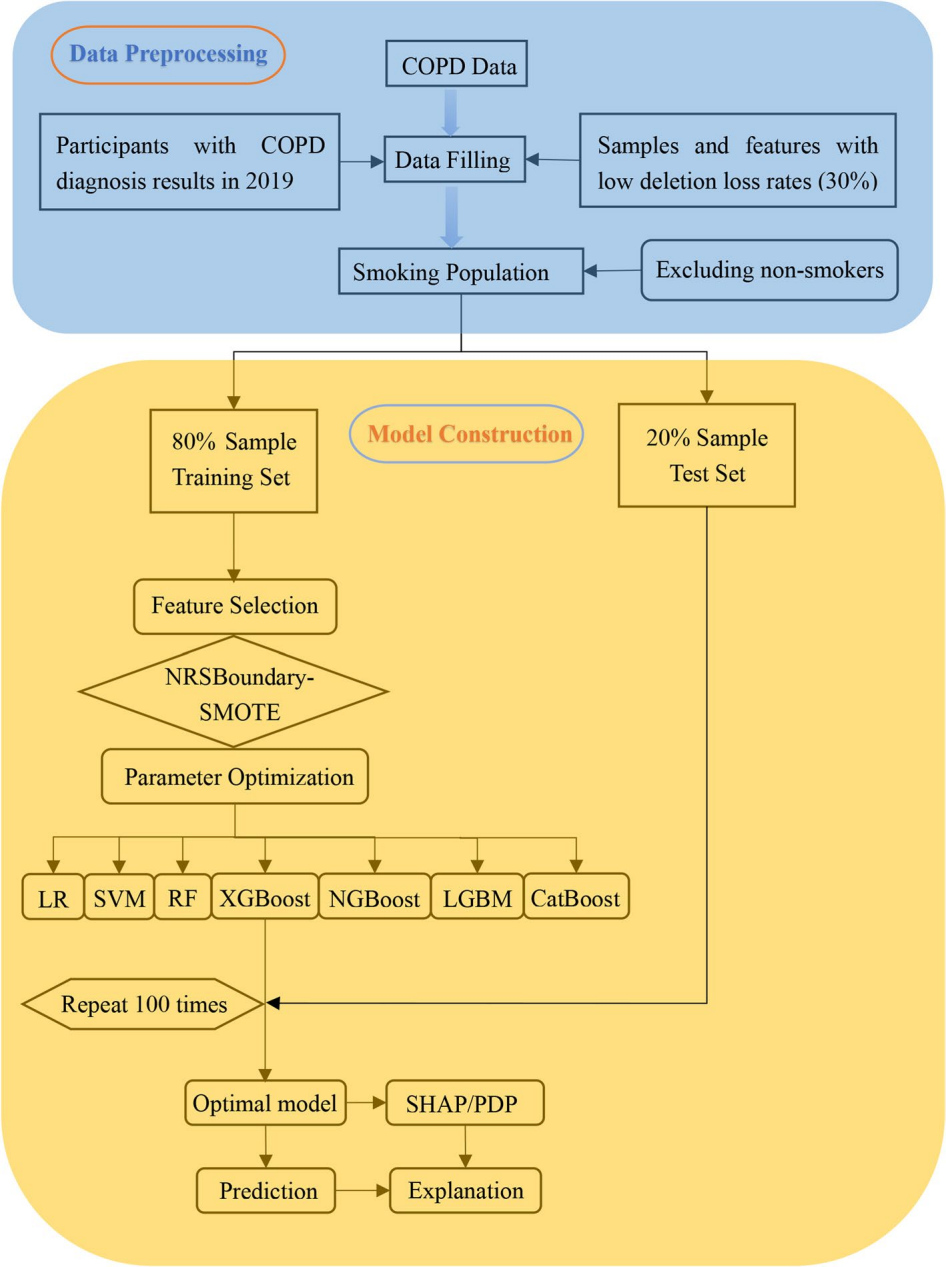
Flow chart of model development and evaluation

To construct and compare all models, several phases were completed. First, we used Boruta to perform feature selection in the sample dataset, acquiring a new reduced dataset for each of sample. Second, the new dataset introduced seven classifiers, namely, CatBoost, NGBoost, XGBoost, LGBM, RF, LR, and SVM, for generating predictions. A grid search method with fivefold CV was performed on the training data to determine the optimal hyperparameters of the above models. Third, the feature-screened dataset was balanced using the NRSBoundary-SMOTE resampling technique, and the seven classifiers mentioned above were then reintroduced to create fresh predictions. Finally, the best performing model out of the seven models was chosen for further model interpretation after a thorough review of multiple evaluation criteria. The entire process is presented in Fig. 1.

The construction and assessment of all models were accomplished through the usage of the stratified hold-out test. To ensure the consistency of the data distribution, stratified sampling was used to split the data into a training set (80%) and a test set (20%) (Tables S5 and S6). Internal verification was performed using the training set, and external verification was performed using the test set. To minimize the statistical variability, the data segmentation and model setting process were repeated 100 times in the training set (the data split ratio was maintained at 8:2). The evaluation of the model performance on the training set was based on the average results of the 100 hold-out tests. In addition, the test set was utilized to confirm the model’s predictive performance to demonstrate the generalization performance of the model. Each model’s performance was evaluated using seven assessment indicators: accuracy, specificity, sensitivity, AUC, F1-score, and G-mean. To ensure the model’s generalizability, all feature selection and data balancing processes were carried out in only the training set, the test set had the same features as the training set, and no processing was performed on the test set data.

### Baseline characteristics

As mentioned above, the data were from 2445 participants, with 15.50% of the sample (387 participants) with COPD. The general characteristics of the study population are presented in Tables S3 and S4. Among the 2445 smokers, 2378 (97.3%) were male and 58 (2.7%) were female. Their average age was 57.28 years. The majority of smokers had a history of second-hand smoke (61.7%) and were current smokers (80.4%). COPD was more prevalent in rural areas (17.3%) than in urban areas (12.2%).

### Univariate analysis

The distribution of COPD patients among the different factors and the results of the univariate analysis are shown in Tables S3 and S4. Univariate analysis involved the chi-square test and nonparametric tests (Mann‒Whitney U test), and the significance threshold was set at 0.10. The findings revealed that there was a statistically significant difference in the prevalence of COPD between the groups (*P* < 0.10) for 21 factors, including occupation, education level, region, sex, age, BMI, family history, central obesity, and CAT scores (see Tables S3 and S4 for details on the other factors).

### Variable selection by Boruta

**Fig. 2:**
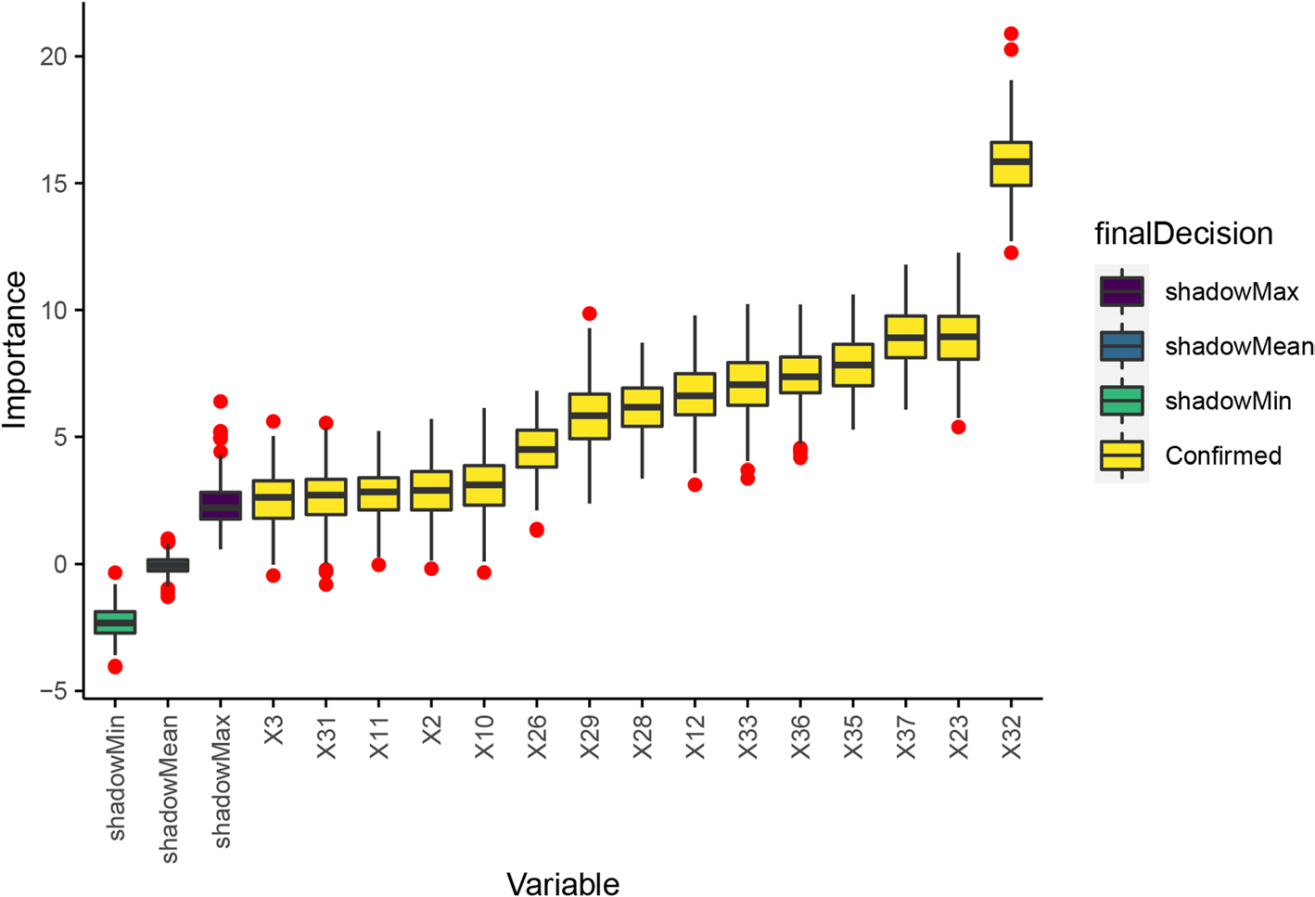
Variable selection using Boruta

To enhance the model’s predictive performance, the Boruta method was adopted to further filter the variables. One hundred iterations of Boruta were carried out to obtain the applicable variables, and the selection results are summarized in Fig. 2. This approach can identify all the applicable features for classification in terms of importance. Out of 21 features, 6 were rejected, and 15 were confirmed.

### Model establishment and evaluation

To minimize statistical variability, the data segmentation and model construction process were repeated 100 times in the training set (the data split ratio was 8:2). The evaluation of model performance in the training set was based on the average results of the 100 stratified hold-out tests. Table 3 summarizes the internal validation of each model in the smoking population dataset, revealing that all models had excellent specificity (0.980–1.00) before balancing the data, but the sensitivity was between 0.00 and 0.07. This result shows that the class imbalance in the study data prevented ML algorithms from successfully identifying COPD patients. The sensitivity of all models was significantly improved after data balancing using the NRSBoundary-SMOTE resampling technique, as were the corresponding F1-score and G-mean values; comparing the performance of different models, we discovered that the data balancing process effectively improved the classification model’s recognition performance for the minority class of samples.

**Table 3:** Means and standard deviations of 100 cross-validation test results in the training set

Model	AUC		Accuracy		Specificity		Sensitivity		F1 score		G-mean	
	Mean	St. dev	Mean	St. dev	Mean	St. dev	Mean	St. dev	Mean	St. dev	Mean	St. dev
SVM	0.580	0.040	0.842	0.000	1.000	0.000	0.000	0.000	0.000	0.000	0.000	0.000
LR	0.697	0.034	0.846	0.004	0.997	0.003	0.039	0.020	0.073	0.036	0.187	0.061
XGBoost	0.687	0.025	0.836	0.007	0.980	0.008	0.070	0.027	0.117	0.041	0.256	0.051
RF	0.705	0.034	0.844	0.004	0.997	0.003	0.035	0.022	0.066	0.040	0.175	0.069
NGBoost	0.701	0.032	0.843	0.004	0.996	0.003	0.026	0.019	0.049	0.034	0.142	0.074
LightGBM	0.711	0.029	0.844	0.004	0.997	0.004	0.030	0.023	0.056	0.041	0.148	0.088
CatBoost	0.712	0.031	0.845	0.005	0.995	0.004	0.041	0.023	0.077	0.041	0.190	0.071
S-LR	0.687	0.032	0.648	0.029	0.659	0.036	0.590	0.065	0.347	0.033	0.622	0.034
S-SVM	0.704	0.034	0.675	0.021	0.688	0.024	0.608	0.059	0.372	0.031	0.646	0.032
S-RF	0.663	0.035	0.736	0.024	0.800	0.029	0.397	0.066	0.322	0.045	0.561	0.046
S-NGB	0.684	0.033	0.710	0.026	0.755	0.032	0.475	0.063	0.342	0.038	0.597	0.039
S-LGB	0.681	0.032	0.673	0.027	0.700	0.033	0.534	0.069	0.341	0.037	0.610	0.039
S-XGB	0.682	0.035	0.645	0.032	0.658	0.036	0.576	0.061	0.340	0.033	0.615	0.034
S-CAT	0.687	0.030	0.706	0.022	0.745	0.029	0.500	0.059	0.350	0.033	0.609	0.034

*S-LR* Logistic regression with NRSBoundary-SMOTE, *S-SVM* SVM with NRSBoundary-SMOTE, *S-RF* Random forest with NRSBoundary-SMOTE, *S-NGB* NGBoost with NRSBoundary-SMOTE, *S-LGB* LightGBM with NRSBoundary-SMOTE, *S-XGB* XGBoost with NRSBoundary-SMOTE, *S-CAT* CatBoost with NRSBoundary-SMOTE

In terms of model comparison, the LR, XGBoost, and CatBoost models all performed well in unbalanced datasets. After balancing the data, the SVM model with NRSBoundary-SMOTE had the highest sensitivity (0.608), AUC (0.704), F1 (0.372), and G-mean values (0.646); the RF model with NRSBoundary-SMOTE had the highest accuracy (0.736) and specificity (0.800). When comprehensive metrics were employed as the criterion for model comparison, the SVM model with NRSBoundary-SMOTE performed the best. Furthermore, the LR and CatBoost models with NRSBoundary-SMOTE exhibited good classification performance.

**Fig. 3:**
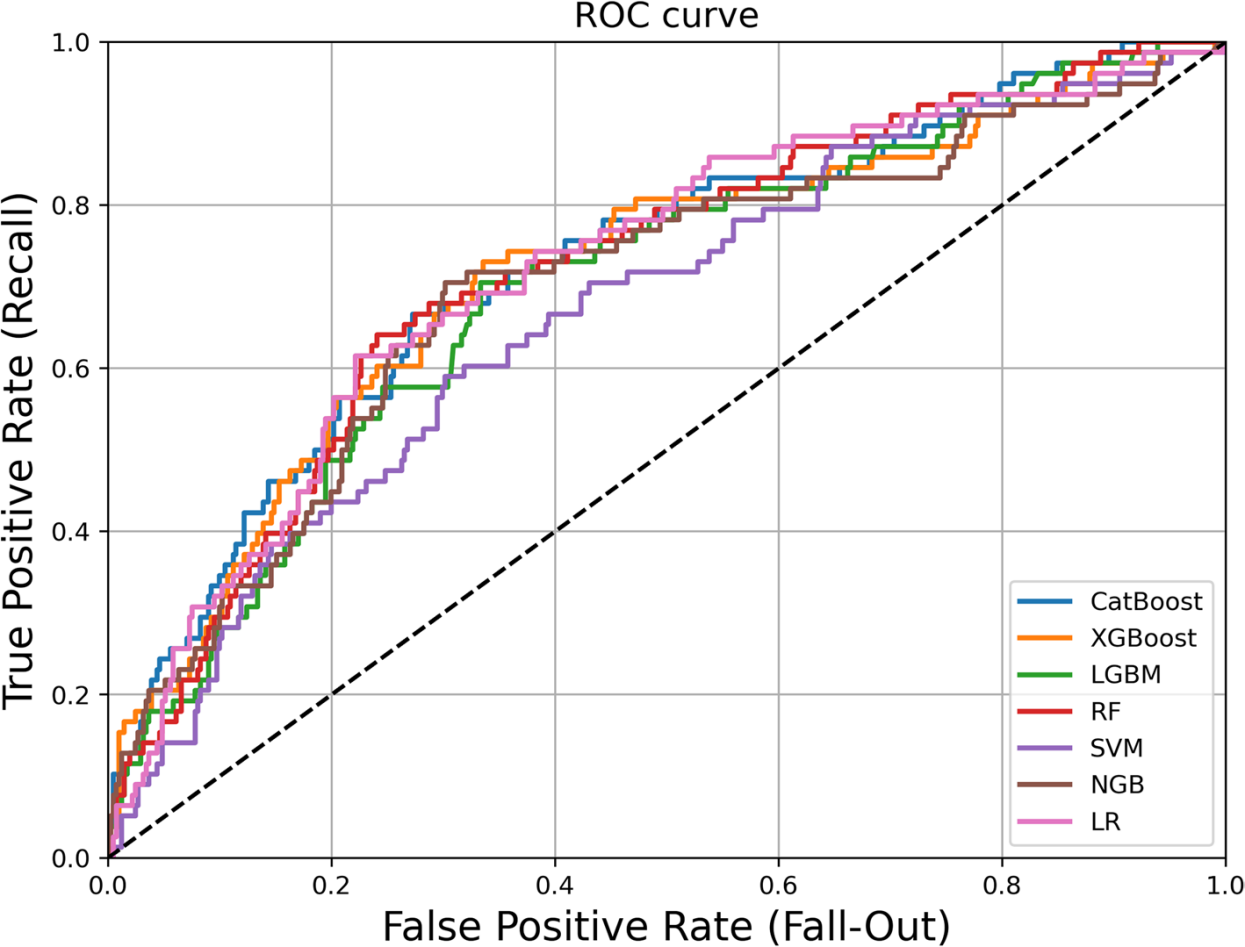
The area under the receiver operating characteristic curve for different prediction models with balanced data

The test set in this study was used for external validation of each model to confirm its generalizability, and the findings (Table 4 and Fig. 3) showed that the predictive performance of models was largely compatible with that of the internal validation. Of the models, the XGBoost model achieved the highest sensitivity, F1 score, and G-mean values in the unbalanced dataset’s external validation results, as well as high values of the AUC, accuracy, and specificity with the best predictive performance. After data balancing, the CatBoost model with the NRSBoundary-SMOTE resampling technique produced the highest AUC (0.727), F1-score (0.425), and a relatively high G-mean (0.669), while the XGBoost and RF models with the NRSBoundary-SMOTE resampling technique achieved the highest specificity (0.808). The maximum sensitivity value (0.628) and highest G-mean value (0.683) were attained by the SVM and NGBoost models with NRSBoundary-SMOTE. When the comprehensive metric was employed as the criterion for model comparison, the CatBoost model with the NRSBoundary-SMOTE resampling technique achieved the best classification performance. The SVM model, which performed best in the training set, did not achieve the best classification performance in the test set, as the CatBoost model generalized better than the SVM model.

**Table 4:** Summary of model performance for external validation data

Model	AUC	Acc	Specificity	Sensitivity	F1 score	G-mean
SVM	0.600	0.841	1.000	0.000	0.000	0.000
LR	0.724	0.841	0.993	0.039	0.071	0.195
XGB	0.658	0.834	0.983	0.051	0.090	0.225
RF	0.713	0.843	0.995	0.039	0.072	0.196
NGB	0.687	0.839	0.993	0.026	0.048	0.160
LGB	0.705	0.836	0.995	0.000	0.000	0.000
CAT	0.718	0.841	0.993	0.039	0.071	0.195
S-LR	0.724	0.732	0.681	0.615	0.423	0.681
S-SVM	0.717	0.695	0.708	0.628	0.397	0.667
S-RF	0.721	0.757	0.808	0.487	0.390	0.627
S-NGB	0.700	0.724	0.742	0.628	0.421	0.683
S-LGB	0.701	0.687	0.708	0.577	0.370	0.639
S-XGB	0.717	0.785	0.808	0.436	0.393	0.593
S-CAT	0.727	0.757	0.793	0.564	0.425	0.669

*S-LR* Logistic regression with NRSBoundary-SMOTE, *S-SVM* SVM with NRSBoundary-SMOTE, *S-RF* Random forest with NRSBoundary-SMOTE, *S-NGB* NGBoost with NRSBoundary-SMOTE, *S-LGB* lightGBM with NRSBoundary-SMOTE, *S-XGB* XGBoost with NRSBoundary-SMOTE, *S-CAT* CatBoost with NRSBoundary-SMOTE

### Visualization of feature importance

**Fig. 4:**
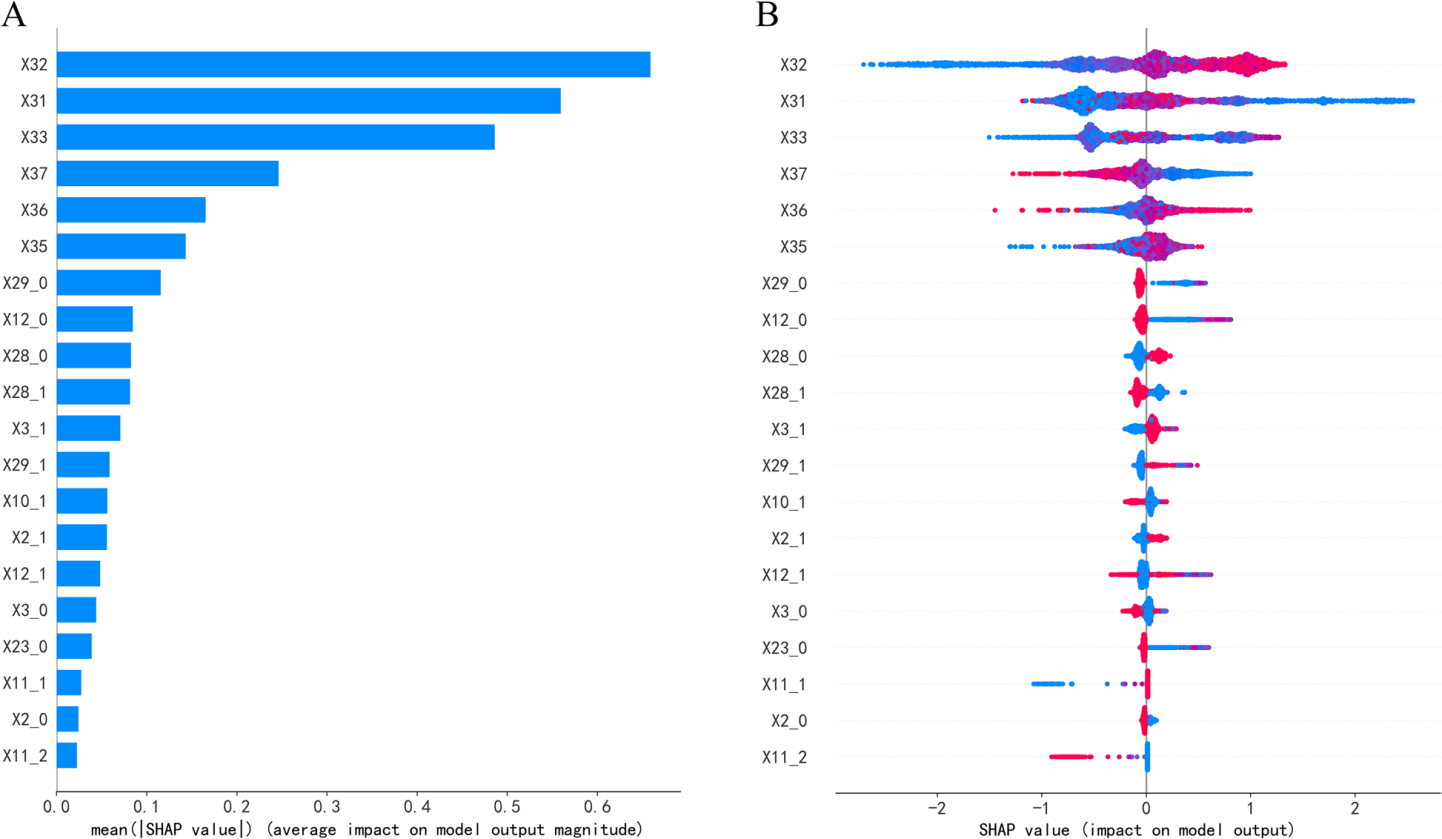
Interpretation of the CatBoost model. **A** SHAP overall feature importance chart. **B** Distribution of characteristic Shapley values

Figure 4A and B show the Shapley value plots. Figure 4A shows the overall feature Shapley value plot, which illustrates the absolute importance of each feature for the model prediction results. Figure 4B displays the typical Shapley values for each sample. The colours represent the magnitude of the highlighted values, while the horizontal coordinates represent the Shapley values. Red dots indicate a high-risk value, whereas blue dots indicate a low-risk value. The irregularly overlapping points explain the dispersion.

As shown in Fig. 4A-B, age was the most significant risk factor for COPD in the smoking population; the older a person was, the more likely they were to have the disease. The CAT score was the second leading risk factor, and the other factors (in descending order) were gross annual income, BMI, SBP, DBP, etc. Furthermore, it is clear from Fig. 4B that “central obesity”, “higher BMI”, and “female sex” had negative SHAP values (i.e., negative associations with COPD). It is straightforward that female smokers with higher BMI values and central obesity have a lower risk of developing COPD.

### Impact of individual features on prediction

Based on the previous ranking of feature importance, we identified six variables (X_32_, X_31_, X_33_, X_37_, X_36_, and X_35_) with the greatest impact on predictions. These variables were as follows: participant age, CAT scores, total annual income of the household, body mass index (BMI), systolic blood pressure (SBP), and diastolic blood pressure (DBP). These six indicators encompass various dimensions, including the age of the participants, their economic status (total annual household income), their basic physical condition (BMI, SBP, and DBP), and the influence of COPD-related symptoms on their lives (the CAT assesses symptoms such as coughing, sputum production, chest tightness, sleep, energy, mood, and activity levels). Therefore, using these six critical influencing factors as examples, we used the PDP method to elucidate the impact of these factors on model predictions.

**Fig. 5:**
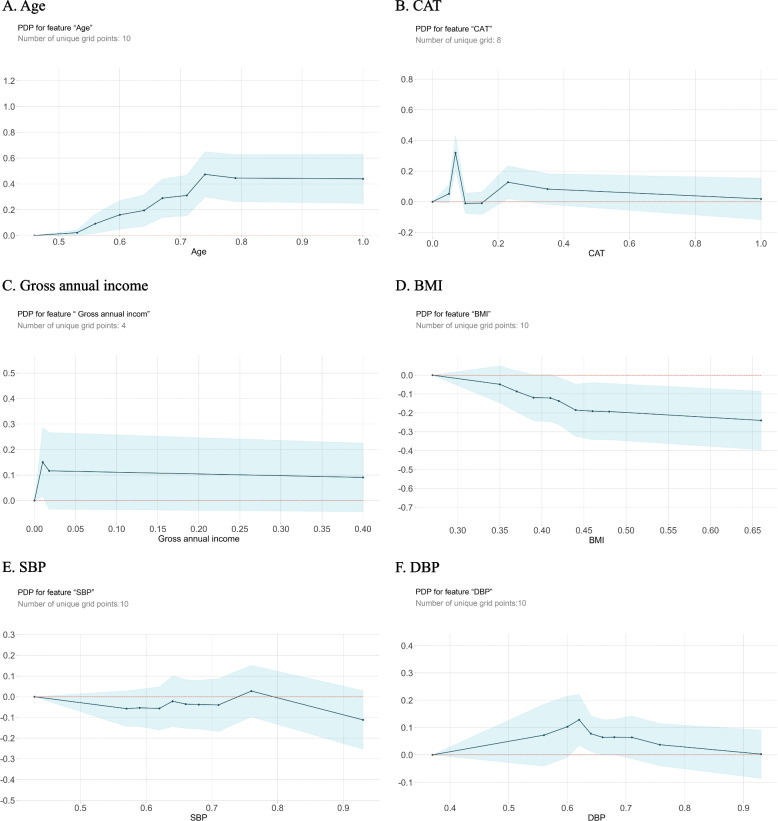
PDP diagram of important variables in the CatBoost model. Note: The y-axis values represent the probabilities of disease risk predicted by the CatBoost model for participants; the x-axis values represent the specific values after variable normalization, which correspond one-to-one with the unnormalized variable values

As shown in Fig. 5, partial dependency plots for age, CAT scores, gross annual income, BMI, SBP, and DBP were generated to analyse the influence of these six characteristics on predicted COPD risk. The y-axis is the magnitude of the change predicted by the model, and it represents the mean value of the prediction, which is based on the leftmost number of the x-axis; the graphs were generated with 0 as the prediction base. The x-axis represents the variation in each independent variable, and the light blue shaded area represents the confidence interval; the larger the interval is, the greater the range of predicted results. The graph demonstrates that the older the person, lower the BMI had a greater impact on the predicted outcome and increased the likelihood of developing COPD. This result supports the SHAP-derived conclusions above. The impacts of gross annual income, SBP, and DBP on model predictions had an overall rising and then falling trend, with multiple turning points in the CAT score, i.e., an upwards trend for CAT scores of 0–2 points, a downwards trend for CAT scores of 2–4 points, a rise for CAT scores of 4–6 points, and a downwards trend for CAT scores of 6 points and over. Partial dependence plots can reveal the relationship between the features and the model predictions, which in turn helps us understand the model prediction results.

### Impact of two features on prediction

**Fig. 6:**
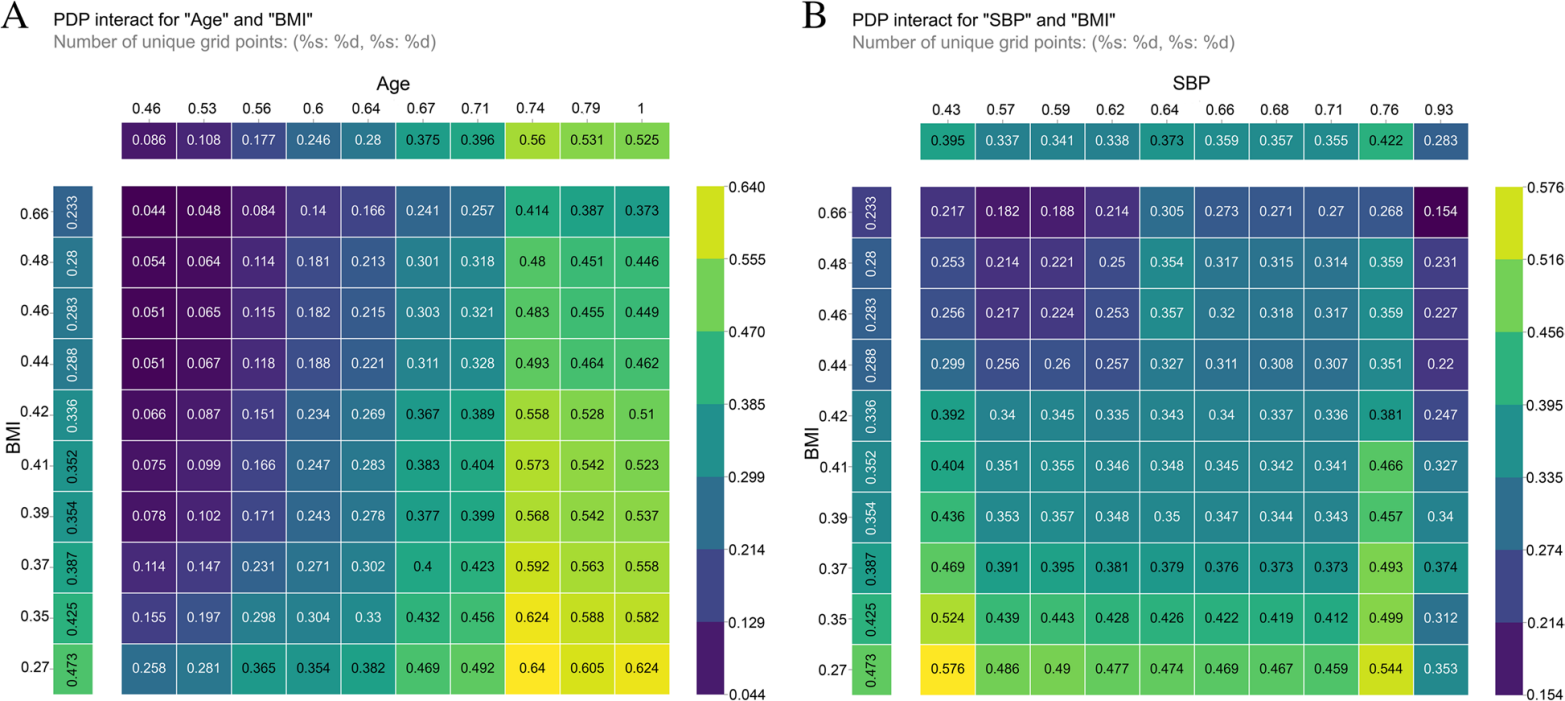
Impact of two features (age and SBP with BMI) on predictions. **A** Effect of age and BMI on predictions. **B** Effect of SBP and BMI on predictions

When considering the impact of individual factors on the prediction results, it is also necessary to consider the joint impact of two factors, i.e., the synergistic effect of the two characteristics on the prediction. Figure 6 shows a heatmap of the effect of two variables on the model’s prediction, with the horizontal and vertical axes showing the variation in the two characteristics, and the third dimension represented by the colour. The lighter the yellow in a region is, the greater the joint impact of the two characteristics on the prediction, and the darker the purple in a region is, the lower its influence on the prediction. According to the joint effect of the two characteristics, decreasing BMI with increasing age had a greater effect on the prediction. Values of SBP that were too low or too high and values of BMI that were lower had a greater impact on prediction.

### Personalized prediction interpretation

**Fig. 7:**
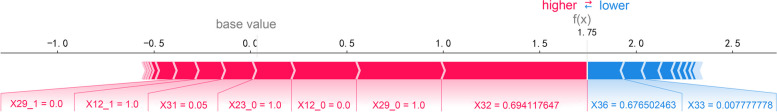
SHAP explanation plot for a patient from our testing dataset

Model predictions for particular patients can be effectively explained and clarified using SHAP values, which show how each feature affected the final forecast. To demonstrate the model’s interpretability, we used a typical example: a 65-year-old man with COPD (Fig. 7). The blue arrows in the figure indicate that a feature will decrease the probability of the sample being classified as COPD, while the red arrows represent a feature that will make it more likely that the sample will be classified as COPD. The width of each arrow indicates the magnitude of the effect of this feature. For the representative patient, age was the feature with the greatest contribution, and it increased the probability that the sample would be predicted to have COPD, that is, older men who smoke are at risk of COPD. The following features with the greatest contributions were anhelation and respiratory disease, where anhelation = 0 and respiratory disease = 1 increased the risk of COPD.

## Discussion

Because smoking-related diseases have high social and medical costs, it is critical to identify and treat these patients early to prevent them from progressing to more severe and expensive stages [60]. The most effective smoking cessation therapies should be made available to people who have or may develop COPD, according to a recent consensus [61]. According to the consensus, identifying patients as early as feasible in the course of the disease can help to prevent smoking and maximize quitting.

Given the above findings, this study aimed to identify individuals at risk for COPD as early as possible by developing an explainable artificial intelligence framework based on COPD surveillance data from the smoking population, as well as to investigate the risk factors for COPD in the smoking population. We investigated various machine learning methods for classifying data in datasets with class imbalance that combined FAMD, NRSBoundary-SMOTE, and Boruta. SHAP and PDP were used to investigate the interpretability of the model predictions.

The study’s findings revealed that the balanced dataset derived with the NRSBoundary-SMOTE oversampling method led to a significant improvement in the model’s predictive performance, especially in the values of indicators such as sensitivity, F1-score and G-mean. In particular, the SVM model, which is more sensitive to unbalanced data, was honed significantly after the balancing process (sensitivity increased from 0 to 0.62). Therefore, there is a strong need for appropriate data balancing techniques to reduce the impact of imbalance. In particular, the performance of the SVM model, which was more sensitive to unbalanced data, was significantly improved after data balancing (the sensitivity increased from 0 to 0.62). Therefore, appropriate data balancing techniques are urgently needed to reduce the impact of imbalance. In the comparison of model performance, we found that the more advanced ensemble model, CatBoost, achieved the highest AUC, accuracy, and F1-score values among the seven ML classifiers, which is consistent with the findings of a previous study. For example, Kim et al. used various machine learning algorithms and the SHAP explanation method to predict acute central vertigo using simple clinical data, and CatBoost had the greatest AUROC values of the ML models tested (0.738) [62], which is consistent with the findings of this study. Additionally, in other disease studies, Kang EA et al. [63] and Mohanty SD et al. [64] reached similar conclusions. The superiority of the CatBoost model has been clearly demonstrated. However, due to the intricacy of clinical decision-making, it is frequently more persuasive to combine suitable data preprocessing techniques with multiple interpretation techniques. In contrast to Kim’s (2021) prescience system, we emphasize the integration of appropriate data preprocessing methods, various complex models, and interpretable methodologies to increase the clinical understanding of COPD risk in the smoking population.

We further identified important COPD risk factors and determined how these variables influenced the CatBoost model’s decision-making processing using SHAP and PDP. According to our findings, the most important factors for predicting COPD in the smoking population were age, CAT scores, gross annual income, BMI, anhelation, respiratory disease, central obesity, use of polluting fuel for household heating, region, use of polluting fuel for household cooking, and wheezing. This is similar to findings in previous research [5, 65–70]. SBP and DBP were significant predictors of COPD in the current study, which may be related to the predisposition of COPD patients to cardiovascular disease, which is consistent with the findings of Johnston et al. [71]. In terms of the interpretability of the model’s decision-making process, when the classification model identifies individuals as being at high risk of COPD, health care professionals can gain insights from interpretability analysis regarding the factors that contributed to their classification as high-risk individuals. Clinicians can thus understand the high-risk factors specific to an individual and the relative importance of multiple predictive factors in determining the final model prediction. This helps to provide a better understanding of the decision-making process of the screening model, similar to the explanation provided in Fig. 7 of the paper’s results: factors such as an age of 65, breathlessness, respiratory conditions, wheezing, and a CAT score of 14 (moderate impact) are the primary reasons that the model identified this individual as being at high risk of COPD, with these variables listed in decreasing order of their contribution (as indicated by the width of the red bars in Fig. 7). In summary, medical professionals can make more informed decisions with the support of the comprehensive information presented in the results and interpretations of risk factors rather than just believing the algorithm’s prediction. Additionally, local explanations might assist medics in comprehending why the model suggests particular actions for individuals classified as high risk. Such subject-by-subject prediction breakdowns have the potential to personalize prevention.

Our research had some limitations. First, this study’s predictors included only questionnaire information and simple physical measurements from COPD surveillance data, but no lung function monitoring data were included, resulting in a relatively low COPD identification rate. Second, an independent dataset should have been used to provide external validation of our work, demonstrating the superiority of our model. Furthermore, deep learning has reportedly been utilized to create medical models as artificial intelligence has progressed. We intend to create a deep learning model to predict COPD in the future and to combine larger amounts of data and information for various levels of research.

## Conclusion

In this study, we created an explainable artificial intelligence framework by combining data preprocessing methods (FAMD, NRSBoundary-SMOTE, and Boruta), machine learning methods, and SHAP/PDP interpretation methods. The results indicated that a combination of appropriate data preprocessing methods, CatBoost models, and SHAP/PDP can provide a global and local interpretation of model predictions of people at risk for COPD in the smoking population while retaining good predictive performance. It can provide medical practitioners with a more intuitive understanding of the impact of important factors in the model on model prediction, allowing them to better comprehend the decision-making process used to identify high-risk individuals.

### Supplementary Information

**Additional file 1: Supplementary Table S1.** Sampling process of survey subjects for COPD surveillance in China. **Supplementary Table S2.** Parameter setting. **Supplementary Table S3.** Detection rate of COPD with categorical variable of different populations. **Supplementary Table S4.** Detection rate of COPD with continuous variable of different populations. **Supplementary Table S5.** Sample situation. **Supplementary Table S6.** Distribution of train/test data.

## Data Availability

The data that support the findings of this study are available from the corresponding author upon reasonable request.

## Abbreviations

COPD: Chronic obstructive pulmonary disease
CAT: COPD Assessment Test
BMI: Body Mass Index
SBP: Systolic blood pressure
DBP: Diastolic blood pressure
FAMD: Factorial analysis for mixed data
ML: Machine learning
SVM: Support vector machine
LR: Logistic regression
RF: Random forest
XGBoost: Extreme gradient boost
LightGBM: Light gradient boosting machine
NGBoost: Natural gradient boosting
CatBoost: Category boosting
ML: Machine learning
SHAP: SHapley Additive exPlanations
PDP: Partial Dependence Plot
