# Supplementary material for: An explainable artificial intelligence framework for risk prediction of COPD in smokers
Source: BMC Public Health. 2023 Nov 6;23:2164. doi: 10.1186/s12889-023-17011-w (PMC10626705; doi:10.1186/s12889-023-17011-w)
Supplement: Supplementary file 1 — Additional file 1: Supplementary Table S1. Sampling process of survey subjects for COPD surveillance in China. Supplementary Table S2. Parameter setting. Supplementary Table S3. Detection rate of COPD with categorical variable of different populations. Supplementary Table S4. Detection rate of COPD with continuous variable of different populations. Supplementary Table S5. Sample situation. Supplementary Table S6. Distribution of train/test data. [file 12889_2023_17011_MOESM1_ESM.docx]

**An** **Explainable Artificial Intelligence Framework** **for risk prediction of COPD in smokers**

**Xuchun Wang^a^, Yuchao Qiao^a^, Yu Cui^a^, Hao Ren^a^, Ying Zhao^b^, Liqin Linghu^a,b^, Jiahui Ren^a^, Zhiyang Zhao^a^, Limin Chen^c,**^, Lixia Qiu^a,*^**

^a^ Department of Health Statistics, School of Public Health, Shanxi Medical University, Taiyuan, Shanxi, 030001, China

^b^ Shanxi Centre for Disease Control and Prevention, Taiyuan, Shanxi, 030012, China

^c^ The Fifth Hospital (Shanxi People’s Hospital) of Shanxi Medical University, Taiyuan, Shanxi, 030012, China

**Co-corresponding author: Limin Chen, The Fifth Hospital (Shanxi People’s Hospital) of Shanxi Medical University, Taiyuan, Shanxi, 030012, P.R.China

Phone: +86-13934519150

E-mail: [sxchenlimin@163.com (LMC)](mailto:sxchenlimin@163.com%20(LMC)) ;

* Corresponding author: Lixia Qiu, Department of Health Statistics, School of

Public Health, Shanxi Medical University, 56 South XinJian Road, Taiyuan, 030001, P.R.China

Phone: +86-13453189351

E-mail: [qlx_1126@163.com(LXQ)](mailto:qlx_1126@163.com(LXQ)) ;

1. **Sampling method and process**

In order to make the COPD surveillance data more representative, four national monitoring sites in Shanxi Province (Qingxu in Taiyuan city, Lingqiu in Datong City, Yuanping in Xinzhou City, Houma in Linfen City) were selected. Additionally, seven monitoring sites (Pingding in Yangquan City, Huguan in Changzhi City, Zezhou in Jincheng City, Shuozhou in Shouzhou City, Yuci in Jinzhong City, Wanrong in Yuncheng City, Linxian in Luliang City) were expanded and related projects were investigated.

A multi-stage stratified whole-group sampling method was used to randomly select three townships/streets in each monitoring site; two administrative villages/residential committees were randomly selected in each sampled township/street; one villager/residential group was randomly selected in each sampled administrative village/residential committee, with each villager/residential group containing at least 150 villagers/residents; 100 households containing residents aged 40 and above were randomly selected as survey households in each sampled villager/residential group; among the sampled survey households, one resident aged 40 and above was randomly selected for the survey using the Kish table method.

| Sampling stage | Sample allocation | Sampling method |
| --- | --- | --- |
| The first stage | Select 3 townships / streets | Probability proportional to size  (PPS) |
| The second stage | Select 2 administrative villages / residential committees | Probability proportional to size  (PPS) |
| The third stage | Select 1 villager / residential group (at least 150 villagers/residents) | Cluster random sampling |
| The fourth stage | Select 100 households (including residents aged 40 and above) | Simple random sampling |
| The final stage | One resident is randomly selected from each household | Kish table |

Supplementary Table S1 Sampling process of survey subjects for COPD surveillance in China

1. **Survey content and method**

(1) Questionnaire survey: The questionnaire included the demographics, disease awareness and knowledge, respiratory symptoms, disease management, risk factors like smoking, indoor polluting fuel exposure, workplace exposure to dust or chemicals, and pulmonary function test contraindications. (2) Anthropometric measurements include height, weight, waist circumference, hip circumference, and blood pressure. (3) Pulmonary function tests: All participants in this surveillance were required to undergo pulmonary function tests to determine their pulmonary function and the presence of persistent airway obstruction. The measurement indicators mainly include forced expiratory volume in one second (FEV1), forced expiratory volume in six seconds (FEV6), and forced vital capacity (FVC), among others. The survey participants first performed the basic pulmonary function test, then the bronchodilator test, inhaled 400 micrograms of the bronchodilator salbutamol aerosol, and repeated the pulmonary function measurement 15 minutes later. For subjects with airway obstruction on pulmonary function tests (FEV1/FVC 70%), an anterior chest X-ray was taken.

1. **Definitions**

(1) Chronic obstructive pulmonary disease (COPD): Referring to the Global Initiative for Chronic Obstructive Pulmonary Disease (GOLD), patients with COPD were diagnosed with a pulmonary function index of FEV1/FVC <0.70 after a bronchodilatation test[1]. (2) Smoking exposure: Survey respondents had daily or occasional active smoking behavior at the time of the survey. (3) Secondhand smoke exposure: The number of days per week that a survey respondent is exposed to tobacco and smoke emitted from the end of a cigarette by an active smoker is greater than or equal to 1 day. (4) Central obesity refers to male waist circumference≥85cm, female waist circumference ≥80cm[2]. (5) Household air pollution: Referred the use of wood, animal manure or coal for cooking or heating over the past six months or more. (6) Exposure to dust or harmful gases at work (including farm work) was defined as occupational exposure. (7) One or both parents who had suffered from respiratory diseases such as asthma, chronic bronchitis, emphysema, were defined as having a family history of respiratory diseases. COPD Assessment Test (CAT)[3]: The CAT score is obtained by assessing the impact of pulmonary disease on the study subjects, including their cough, sputum production, chest tightness, sleep quality, energy levels, mood, and activity capacity. This score encompasses pulmonary disease-related symptoms and the emotional and activity aspects of the study subjects themselves.

1. **Prediction models**

| Models | Hyperparametric | Final value |
| --- | --- | --- |
| LR | L | L2 |
|  | C | 0.156 |
| SVM | Gamma | 0.025 |
|  | C | 4 |
|  | kernel | ‘rbf’ |
| RF | n_estimators | 300 |
|  | max_features | 6 |
| XGBoost | n_estimators | 150 |
|  | learning_rate | 0.02 |
|  | max_depth | 6 |
| Lightgbm | n_estimators | 400 |
|  | learning_rate | 0.01 |
|  | max_depth | 2 |
| NGBoost | n_estimators | 500 |
|  | learning_rate | 0.01 |
| CatBoost | n_estimators | 500 |
|  | learning_rate | 0.01 |
|  | max_depth | 4 |

Supplementary Table S2 Parameter setting

1. **Univariate Analysis**

| factors | level | num | COPD | rates (%) | *χ^2^* | *P* |
| --- | --- | --- | --- | --- | --- | --- |
| Occupation | Agricultural | 1310 | 241 | 18.4 | 18.243 | ＜0.001 |
|  | Nonagricultural worker | 1001 | 121 | 12.1 |  |  |
|  | Retired | 134 | 26 | 19.4 |  |  |
| Education level | Elementary school and below | 811 | 164 | 20.2 | 17.240 | ＜0.001 |
|  | Junior and senior high school | 1564 | 214 | 13.7 |  |  |
|  | College degree and above | 70 | 10 | 14.3 |  |  |
| Marital status | Single | 63 | 13 | 20.6 | 1.130 | 0.568 |
|  | Married or cohabiting | 2244 | 354 | 15.8 |  |  |
|  | Divorced, widowed or separated | 138 | 21 | 15.2 |  |  |
| Region | Rural | 1750 | 303 | 17.3 | 9.631 | 0.002 |
|  | Urban | 695 | 85 | 12.2 |  |  |
| Sex | Male | 2387 | 385 | 16.1 | 5.092 | 0.024 |
|  | Female | 58 | 3 | 5.2 |  |  |
| Cough | No | 2211 | 324 | 14.7 | 25.549 | ＜0.001 |
|  | Yes | 234 | 64 | 27.4 |  |  |
| Productive cough | No | 2007 | 296 | 14.7 | 10.540 | 0.001 |
|  | Yes | 438 | 92 | 21.0 |  |  |
| Wheezing | No | 2290 | 329 | 14.4 | 61.065 | ＜0.001 |
|  | Yes | 155 | 59 | 38.1 |  |  |
| Anhelation | No | 2128 | 288 | 13.5 | 67.044 | ＜0.001 |
|  | Yes | 317 | 100 | 31.5 |  |  |
| Premature birth | No | 2372 | 373 | 15.7 | 1.234 | 0.267 |
|  | Yes | 73 | 15 | 20.5 |  |  |
| Hospitalization for pneumonia or bronchitis at or before the age of 14 | No | 2414 | 383 | 15.9 | 0.002 | 0.968 |
|  | Yes | 31 | 5 | 16.1 |  |  |
| Hospitalization for pneumonia or bronchitis between the ages of 15 and 17 | No | 2434 | 384 | 15.8 | 3.476 | 0.062 |
|  | Yes | 11 | 4 | 36.4 |  |  |
| Respiratory disease | No | 2150 | 300 | 14.0 | 48.979 | ＜0.001 |
|  | Yes | 295 | 88 | 29.8 |  |  |
| Malignant tumour | No | 2438 | 387 | 15.9 | 0.014 | 0.907 |
|  | Yes | 7 | 1 | 14.3 |  |  |
| Cardiovascular disease | No | 1763 | 276 | 15.7 | 0.217 | 0.642 |
|  | Yes | 682 | 112 | 16.4 |  |  |
| Lung surgery | No | 2433 | 384 | 15.8 | 2.754 | 0.097 |
|  | Yes | 12 | 4 | 33.3 |  |  |
| Diabetes mellitus | No | 2317 | 364 | 15.7 | 0.840 | 0.359 |
|  | Yes | 128 | 24 | 18.8 |  |  |

Supplementary Table S3 Detection rate of COPD with categorical variable of different populations

| Depression | No | 2435 | 385 | 15.8 | 1.253 | 0.263 |
| --- | --- | --- | --- | --- | --- | --- |
|  | Yes | 10 | 3 | 30.0 |  |  |
| Osteoporosis | No | 2384 | 376 | 15.8 | 0.678 | 0.410 |
|  | Yes | 61 | 12 | 19.7 |  |  |
| Gastroesophageal reflux | No | 2398 | 382 | 15.9 | 0.346 | 0.557 |
|  | Yes | 47 | 6 | 12.8 |  |  |
| Anaemia | No | 2400 | 382 | 15.9 | 0.221 | 0.638 |
|  | Yes | 45 | 6 | 13.3 |  |  |
| Family history | No | 1938 | 288 | 14.9 | 7.119 | 0.008 |
|  | Yes | 507 | 100 | 19.7 |  |  |
| Second-hand smoke | No | 558 | 110 | 19.7 | 10.906 | 0.004 |
|  | Yes | 1508 | 233 | 15.5 |  |  |
| Current smoking | Unclear | 379 | 45 | 11.9 | ＜0.001 | 0.999 |
|  | No | 479 | 76 | 15.9 |  |  |
|  | Yes | 1966 | 312 | 15.9 |  |  |
| Use of polluting fuel for household cooking | No | 1771 | 231 | 13.0 | 38.421 | ＜0.001 |
|  | Yes | 674 | 157 | 23.3 |  |  |
| Use of polluting fuel for household heating | No | 909 | 107 | 11.8 | 18.200 | ＜0.001 |
|  | Yes | 1536 | 281 | 18.3 |  |  |
| Occupational exposure to dust and/or hazardous chemical gases | No | 1546 | 251 | 16.2 | 0.423 | 0.516 |
|  | Yes | 899 | 137 | 15.2 |  |  |
| Pulmonary function | No | 2297 | 365 | 15.9 | 0.013 | 0.910 |
|  | Yes | 148 | 23 | 15.5 |  |  |
| Awareness of COPD | No | 2153 | 349 | 16.2 | 1.568 | 0.210 |
|  | Yes | 292 | 39 | 13.4 |  |  |
| Central obesity | No | 752 | 158 | 21.0 | 21.504 | ＜0.001 |
|  | Yes | 1693 | 230 | 13.6 |  |  |

Supplementary Table S3 Continued

| factors | Total | COPD | | *Z* | *P* |
| --- | --- | --- | --- | --- | --- |
|  | Median±QR* | NO | YES |  |  |
| Age, years | 57.00±14.00 | 56.00±14.00 | 63.00±12.00 | -10.842 | ＜0.001 |
| BMI | 24.83±4.64 | 24.98±4.57 | 24.22±4.78 | -5.524 | ＜0.001 |
| Heart rate | 75.33±13.67 | 75.33±13.84 | 75.00±13.66 | -0.502 | 0.615 |
| SBP | 133.00±21.67 | 132.67±21.00 | 135.33±23.25 | -2.486 | 0.013 |
| DBP | 79.00±14.00 | 79.33±14.00 | 77.67±13.33 | -2.488 | 0.013 |
| CAT score | 4.00±9.00 | 4.00±8.00 | 6.00±10.00 | -5.873 | ＜0.001 |
| Gross annual income | 20000.00±26000.00 | 20000.00±26000.00 | 14750.00±24000.00 | -3.622 | ＜0.001 |
| Size of the premises | 80.00±61.00 | 90.00±64.00 | 62.50±60.00 | -5.346 | ＜0.001 |

Supplementary Table S4 Detection rate of COPD with continuous variable of different populations

Note: *Quartile Range

1. **Experimental Setup**

| Samples | Num | Proportion（%） |
| --- | --- | --- |
| Total | 2445 | 100 |
| Training set | 1956 | 80 |
| Test set | 489 | 20 |
| True | 387 | 15.8 |
| False | 2058 | 84.2 |

Supplementary Table S5 Sample situation

| Group | Train (%) | Test (%) |
| --- | --- | --- |
| COPD | 310(16.0) | 78(15.9) |
| Non-COPD | 1646(84.0) | 411(84.1) |
| Total | 1956(80.0) | 489(20.0) |

Supplementary Table S6 Distribution of train/test data

**Reference**

1. Feinstein L, Wilkerson J, Salo PM, MacNell N, Bridge MF, Fessler MB, Thorne PS, Mendy A, Cohn RD, Curry MD *et al*: **Validation of Questionnaire-based Case Definitions for Chronic Obstructive Pulmonary Disease**. *Epidemiology (Cambridge, Mass)* 2020, **31**(3):459-466.

2. Huang X, Zhou Z, Liu J, Song W, Chen Y, Liu Y, Zhang M, Dai W, Yi Y, Zhao S: **Prevalence, awareness, treatment, and control of hypertension among China's Sichuan Tibetan population: A cross-sectional study**. *Clinical and experimental hypertension (New York, NY : 1993)* 2016, **38**(5):457-463.

3. Jones PW, Harding G, Berry P, Wiklund I, Chen WH, Kline Leidy N: **Development and first validation of the COPD Assessment Test**. *The European respiratory journal* 2009, **34**(3):648-654.
